# Collagen Type I Biomaterials as Scaffolds for Bone Tissue Engineering

**DOI:** 10.3390/polym13040599

**Published:** 2021-02-17

**Authors:** Gustavo A. Rico-Llanos, Sara Borrego-González, Miguelangel Moncayo-Donoso, José Becerra, Rick Visser

**Affiliations:** 1 Department of Cell Biology, Genetics and Physiology, Faculty of Science, University of Málaga, IBIMA, 29071 Málaga, Spain; garico@bionand.es (G.A.R.-L.); becerra@uma.es (J.B.); 2 Andalusian Center for Nanomedicine and Biotechnology (BIONAND), 29590 Málaga, Spain; sarbogo@gmail.com (S.B.-G.); mmoncayod@unal.edu.co (M.M.-D.); 3 Networking Research Center on Bioengineering, Biomaterials and Nanomedicine (CIBER-BBN), 29071 Málaga, Spain; 4 Materials Science Institute of Seville (ICMS), Joint CSIC-University of Seville Center, 41092 Seville, Spain; 5 Tissue Engineering Group, Department of Pharmacy, Universidad Nacional de Colombia, Bogotá 111311, Colombia; 6 Biomimetics Laboratory, Biotechnology Institute, Universidad Nacional de Colombia, Bogotá 111311, Colombia

**Keywords:** collagen, bone tissue engineering, scaffolds, growth factors, peptides

## Abstract

Collagen type I is the main organic constituent of the bone extracellular matrix and has been used for decades as scaffolding material in bone tissue engineering approaches when autografts are not feasible. Polymeric collagen can be easily isolated from various animal sources and can be processed in a great number of ways to manufacture biomaterials in the form of sponges, particles, or hydrogels, among others, for different applications. Despite its great biocompatibility and osteoconductivity, collagen type I also has some drawbacks, such as its high biodegradability, low mechanical strength, and lack of osteoinductive activity. Therefore, many attempts have been made to improve the collagen type I-based implants for bone tissue engineering. This review aims to summarize the current status of collagen type I as a biomaterial for bone tissue engineering, as well as to highlight some of the main efforts that have been made recently towards designing and producing collagen implants to improve bone regeneration.

## 1. Introduction

Bone is one of the tissues with the highest intrinsic ability to repair itself after an injury. However, there are unfavorable scenarios in which an affected bone might not be able to repair itself successfully. Some of these include non-unions, the incidence of which is around 5–10% of the patients that suffer a bone fracture [1], or massive tissue loss as a result of major traumas, infections, or tumor-related bone resections. It is estimated that more than 2 million bone grafts are performed worldwide each year to provide a solution for those cases in which the natural repair of bone is hampered [2].

**Figure 1:**
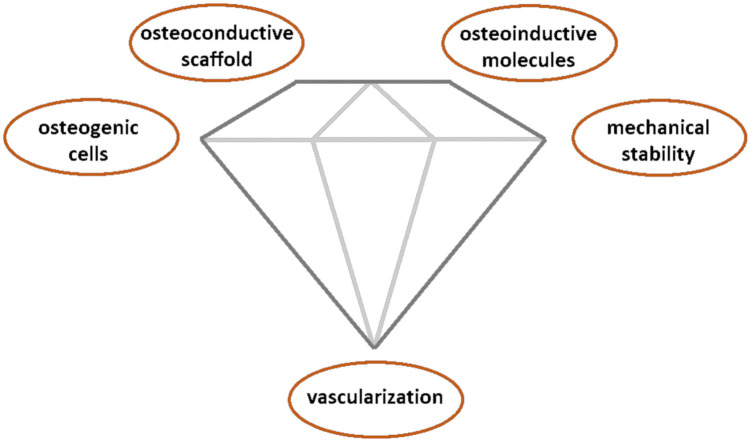
Schematic illustration of the diamond concept, showing the five elements that an ideal bone tissue engineering construct should provide.

Autografts are the current gold standard in orthopedics for the repair of bone defects that are unable to heal spontaneously (the so-called critical bone defects [3] and other interventions, such as bone augmentation or spinal fusion. An autograft provides four biological elements of the so-called diamond concept (Figure 1) of bone healing: osteoinductive and pro-angiogenic molecular signals (growth factors, cytokines and other molecules), osteogenic cells (osteoblasts and skeletal precursors), and an osteoconductive matrix (formed mainly by collagen and hydroxyapatite (HA)). The fifth element is mechanical stability, which must be provided by the physician with help of a variety of external and/or intramedullary fixation devices. The autologous origin of autografts implies that there are no immune rejections or transmission of infections [4,5,6]. However, autografts also have some important drawbacks, such as the limited availability of material for grafting. Furthermore, although the extraction of bone from the iliac crest (the main source of bone for autografting) is a relatively simple procedure, between 8% and 20% of the patients experience one or more complications such as infections, hematoma and seroma formation, secondary fractures, nerve and vascular injuries, chronic pain, or hernias [7,8]. For these reasons, groups dedicated to regenerative medicine and tissue engineering are actively searching for therapeutic alternatives to autografts based on the combination of a biomaterial, osteoprogenitor cells, and/or osteoinductive signals. Besides the clinical relevance, the economic impact for national healthcare systems is enormous; the bone graft substitutes market was valued at 2.4 × 10^9^ US$ in 2015, with a tendency towards doubling this amount in 2025 [9].

A large number of polymeric scaffolds has been explored for bone tissue engineering (BTE). These include both natural polymers, such as fibrin, chitosan, or fibroin, as well as synthetic ones, such as poly(lactic acid) (PLA), poly(glycolic acid) (PGA), or their copolymers poly(lactic co-glycolic acid) (PLGA) [10]. However, since collagen represents the main organic constituent of bone tissue, collagen-based biomaterials have been probably the most used for BTE purposes, as well as for other biomedical applications [11]. This probably places the collagen-based biomaterials among those with a broader biomedical and greater economic interest. 

## 2. Collagen Type I in Bone Tissue Engineering

Collagen is the name used to designate a group of at least 29 different polymeric proteins that, together, are the most abundant protein component of the extracellular matrix (ECM) and represent around 20–30% of the weight of all body proteins in mammals [12]. All the collagen proteins are formed by a characteristic triple helix constituted by three out of all the genetically different pro-collagen polypeptide chains [13]. By far, the most abundant type of collagen is type I, which represents more than 90% of the organic mass of bone and is the major protein constituent of several other tissues like tendons, ligaments, cornea, or skin [13,14]. 

In bone, collagen type I is mainly produced by osteoblasts, which are also responsible for controlling the formation of hydroxyapatite from deposited calcium and phosphate salts. Osseous tissue is highly dynamic, and a continuous, strictly controlled turnover of its ECM occurs in healthy individuals by the osteoresorptive activity of osteoclasts and the osteogenic activity of osteoblasts. During the formation and the breakdown of the ECM, cells can interact with the exposed collagen fibers, which contain specific domains for interacting with cells, such as the well-known, integrin-binding motifs RGD [15] or DGEA [16]. Hence, when used as a scaffolding material in BTE, collagen not only acts as a physical support for cells to attach to and to grow on, but also influences cell behavior and fate through receptor-mediated interactions. These features make collagen type I a widely used biomaterial in tissue engineering applications, either to be used alone or to confer its biological properties to other scaffolds when used as composites [17,18]. 

**Figure 2:**
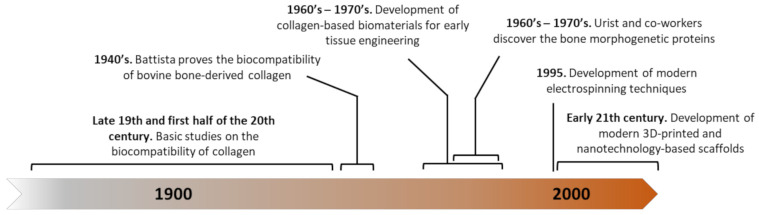
Timeline showing some of the major milestones in the development of collagen-based scaffolds for bone tissue engineering (BTE).

The earliest reports of the use of non-hydrolyzed collagen in the biomedical field date back to the 19th century, through studies to establish its behavior and grafting potential (Figure 2). Examples include the use of decalcified bone for filling bone defects in 1880, collagen isolated from rat tails with acetic acid and grafted into other animals, human cadavers or even living human patients, or collagen isolated from ox cornea and subcutaneously implanted in rabbits. In a more comprehensive study, Battista analyzed the histological reaction to implanting bovine bone-derived collagen (Collatissue A) into different locations, such as subcutaneously, in muscle, peritoneum, the nervous system, and bone [19]. This is one of the first reports on characterizing the cell response to collagen-based implants, observing an initial fibroblastic and monocyte infiltration followed by a gradual degradation until reaching a complete replacement by fibrous tissue.

During the 1960s and 70s, several studies tested the grafting capacity, inflammatory response, degradation rates, vascularization, and other parameters in a great variety of collagen presentations, such as gels, sponges, meshes, or solutions in several models, such as rabbit eyes [20,21], stapedectomies [22], gynecological surgeries [23], or in skeletal defects in rabbit tibiae [24], ribs [25], and osteochondral defects [26], among others. All these studies together established that collagen-based biomaterials: (i) are biocompatible; (ii) have a tunable degradation rate; (iii) does elicit a mild inflammatory response; (iv) is invaded by the host’s cells; and (v) can become vascularized. These features made collagen progressively more popular for enhancing healing processes.

Although, at this point, it was already demonstrated that collagen type I-based biomaterials were suitable and biocompatible, they all failed in promoting successful bone healing when implanted into bone defects. In a series of works between 1965 and 1975, Marshall Urist and co-workers discovered that demineralized bone matrix was able to produce new bone after its subcutaneous or intramuscular implantation [27]. This demonstrated that isolated bone matrix contains osteoinductive biological cues that purified collagen type I lacks. Using different types of digestion, demineralization, and heat denaturation, these authors showed that the osteoinductive properties of bone matrix are related to a protein fraction different than the helical portion of collagen. They named this protein bone morphogenetic protein (BMP) and, with their discovery, opened an entire field in skeletal development and regeneration research [28,29,30,31]. Nowadays it is well known that BMP/transforming growth factor-β (TGF-β) signaling strongly regulates the ossification process during all its phases. Specifically, certain BMPs, such as BMP-2, -4, -6, -7, and -9 have a great osteoinductive effect in different settings, both in vitro and in vivo [32].

## 3. Forms of Collagen Type I Biomaterials for Bone Tissue Engineering

Collagen, either directly extracted from animal tissues, purified, or even produced as recombinant proteins is extremely versatile. Polymerization, fibrillogenesis, and fiber formation are well-known processes and have been modified for obtaining multiple different forms of collagen scaffolds for a great variety of BTE applications. Some illustrative examples have been included in Table 1.

### 3.1. Powders/Particles

One of the most explored forms of collagen in BTE is demineralized bone matrix (DBM) in its powder form. DBM powder is obtained when autologous or allogeneic bone is processed to yield a partially purified bone matrix, devoid of fat and mineral content, which is pulverized until converted into fine grains. A successful application of this type of collagen biomaterial has been in maxillary sinus floor elevation. After the loss of teeth in the maxillary region, the surrounding alveolar bone is markedly resorbed, creating a gap that makes the implantation of tooth implants extremely difficult. In these cases, DBM powders have been used to fill the created gap and to promote the formation of new bone, allowing the implantation of new dental pieces [53,54]. With a similar rationale, purified collagen powders have been used combined with other polymeric and non-polymeric materials as well as with growth factors. Saito and co-workers produced an injectable collagen powder combined with a chimeric, collagen-targeted, recombinant fibroblast growth factor-2 (CBD-FGF-2). These authors demonstrated that their system was able to increase callus volume and bone mineral content in a femoral fracture model in mice [33]. In a more recent study, bovine collagen powder was combined with BMP-2 to successfully enhance bone formation when implanted into vertebral laminar defects created in rabbits [34].

Collagen powder has also been used for increasing the effectiveness of cell therapy strategies. In a recent study, collagen powder was used as a scaffold for administering human umbilical cord-derived mesenchymal stem cells (huMSCs) in an alveolar cleft model in rabbits. The authors reported significantly better bone repair than when implanting the collagen without cells and, when focusing on certain parameters, even than when implanting collagen with BMP-2 [35]. Although they did not include a control group, implanting only cells without the collagen vehicle, it is somewhat predictable that collagen was acting as a scaffold on which cells were retained and induced to differentiate and, thus, increasing osteogenesis.

### 3.2. Fibers and Tubes

It is technically feasible to cast atelocollagen solutions in or around inert molds with a great variety of shapes to manufacture different types of biomaterials. Collagen tubes have been formed around silicone rods with the aim of improving nerve regeneration in a rat tibial nerve resection model [36]. Although collagen tubulization did not significantly enhance nerve regeneration, the tubulized repairs most closely resembled unoperated nerves. A later study showed that crosslinking by UV irradiation of collagen tubes promoted more cellular activity and regeneration compared to tubes that had been chemically crosslinked with glutaraldehyde [37]. When combined with bone marrow-derived mesenchymal stem cells (BM-MSCs), collagen tubes supported better regeneration of peripheral nerve fibers across a 3-mm nerve gap, thus demonstrating that these tubes could be used as regenerative guidance in cell therapy strategies [38].

For obvious anatomical reasons, tubular biomaterials are not frequently used in BTE in a direct way. However, more complex techniques, such as electrospinning manufacturing, are inspired in the fabrication of continuous fibers ranging between the nano- and the micro-scale, in a great variety of forms. This technique, which started to gain popularity for the manufacturing of materials for biomedical applications in the early 2000s, allows the production of polymer fibers with a diameter from 3 nm to more than 5 µm using a relatively simple and inexpensive setup. Although the disposition of the fibers is not finely controlled, highly porous membranes can be obtained. The most typical polymeric materials used for electrospinning are synthetic ones, like poly(L-lactic acid) (PLLA), poly(glycolic acid) (PGA), polycaprolactone (PCL), poly(ethylene oxide) (PEO), and poly(vinyl alcohol) (PVA). However, even though care must be taken to avoid protein denaturation during the process, polymers of natural origin can also be used, such as gelatin, silk fibroin, chitosan, and collagen. The theoretical and technical principles of this technique focused on tissue engineering applications and, specifically, on BTE have been extensively reviewed before [55,56].

Several studies have shown a successful production of polymeric collagen-based scaffolds using electrospinning. Collagen-HA membranes [39], alternate dual meshes of PLGA with a collagen-HA dispersion [40], and collagen-polycatecholamines-CaCO_3_ scaffolds [41] displayed very good mechanical properties and low cytotoxicity, and sustained the osteogenic differentiation of different cells types in vitro. More recently, Guo and co-workers showed very promising results using a collagen-chitosan nanofibrous membrane to achieve almost total closure of full-thickness cranial bone defects in rats 8 weeks after implantation [42].

### 3.3. Gels

Hydrogels are very interesting materials, as they have a great ability to absorb water and allow the diffusion of cells and molecules. Since some of their physicochemical properties can be quite easily tailored during their manufacturing process by modifying their composition, crosslinking conditions, or synthetizing technique, they are highly versatile [57]. Although their poor biomechanical properties do not fulfill the requirements that could be considered ideal for BTE, hydrogels have the advantage of being easy to make in quantities and forms that can adapt to the specific needs of the surgeon.

A simple but interesting collagen format are injectable gels, which can be useful for physicians to fill bone defects using less invasive surgical techniques. Several models have been used to test fluid collagen biomaterials and compare these approaches with bone autografts. Minamide and co-workers analyzed collagen gels as cell carriers in vertebral arthrodesis. Therefore, they cultured bone marrow cells in a collagen type I gel for one week and used the whole in a rabbit spinal fusion model. They showed that this combination improved the success rate of fusions and led to the production of new bone tissue of better quality than autografts or the collagen gel without cells [43].

Collagen gels are also very suitable to be used as part of composite biomaterials to improve the biological or physicochemical properties of other polymeric or non-polymeric materials. As examples, collagen gels have been used to load adipose-derived mesenchymal stem cells into a porous poly(lactic-co-glycolic)acid tricalcium phosphate (PLGA-β-TCP) scaffold [44]. These authors showed that ectopic implantation of their composites in rabbits led to an increased and very homogenous calcified cartilage and bone formation compared to when the cells were delivered directly onto the PLGA-β-TCP scaffold without the collagen gel. This format has also been used as a BMP-2 carrier for its delivery into a tendon-bone interface injury model in rabbits [45]. Since collagen has some tendency to retain BMP-2, in vitro experiments showed that these gels were able to retain up to 50% of the loaded BMP-2 after 5 days, while slowly releasing the remaining growth factor for over 28 days. A single direct injection of this BMP-2-loaded gel induced the formation of fibrocartilage and new bone at the tendon-bone interface at 6 weeks after surgery.

In even more recent approaches, collagen gels have been tested as bioinks for 3D printing applications [58]. This technology allows printing of highly specific and detailed architectures and has been used for fabricating various tissue-like geometries, including collagen/hydroxyapatite composites [46], mesh-like structures of collagen containing hydroxyapatite nanorods [47] or even more complex scaffolds consisting of collagen, decellularized extracellular matrix and silk fibroin [48]. All of these scaffolds were demonstrated to be highly tunable, to have appropriate physicochemical properties, and to be biocompatible when tested in vitro.

### 3.4. Sponges

Collagen sponges are, by far, the most widespread and tested form of collagen scaffold. They are usually manufactured by casting an aqueous collagen solution or gel into a mold and then freeze-dried. Changes in freezing regime, vacuum conditions, type of suspension, and presence of different additives or porogens can completely modify the porous structure, interconnectivity, and architecture of the resulting collagen sponge [59,60]. Even though these types of scaffolds have been extensively studied, clinically tested and some of them even globally commercialized, new technology is still being applied nowadays to increase the regenerative potential of collagen sponges. For example, the mechanical strength of a collagen sponge can be increased by adding synthetic polymers during its fabrication. In this line, poly (glycolic acid) fibers were added to collagen type I to produce collagen/PGA discs that significantly enhanced bone healing in calvarial defects in rabbits, even without the addition of growth factors or cells [49].

In more complex models, collagen sponges have been used as basic scaffolds for housing multifactorial elements. In this sense, Al-Ahmady and co-workers designed a collagen scaffold combined with platelet-rich fibrin and nanohydroxyapatite seeded with autologous bone marrow mononuclear cells for bone regeneration in a small clinical study with patients suffering from unilateral alveolar cleft defects. These authors stated that their treatment had reasonable potential as a therapeutic option for alveolar bone cleft defects, but that further evaluation of the long-term effects was needed [50]. Focused on another application, Zhang and co-workers pre-seeded BM-MSCs on collagen sponges and applied cyclic mechanical stretch. Together with stimulation by TGF-β1, this mechanical stimulation synergistically promoted the differentiation of the cells into tenocytes and enhanced tendon regeneration when implanted into a rat Achilles tendon in an in situ repair model [51].

Collagen type I sponge-like scaffolds are also being tested for the delivery of the secreted products (secretome) produced by undifferentiated cells in culture. In one study, cell-derived nanovesicles were obtained by extruding umbilical cord-derived MSCs (ucMSCs) through nanoporous membranes, following a previously-described protocol [61]. These nanovesicles have certain similarities to exosomes but can be produced faster and in larger quantities. The authors loaded these nanovesicles together with BMP-2 on collagen sponges and implanted these in athymic nude mice calvarial defects. Their results showed that these nanovesicles can increase BMP-2-induced osteogenesis as they reported higher bone volumes, and more bone trabeculae and vessel-like structures than in the control implants [52].

Although it is well known that scaffolds designed for BTE should have an interconnected porosity and an appropriate pore size to allow osteoprogenitor cells to colonize the inner regions of the scaffolds and the growth of new blood vessels, it has recently been described that the orientation of the pores can also influence the osteogenic process [62]. With this in mind, we have recently produced and evaluated polymeric collagen type I sponge-like scaffolds with uni- or multidirectional pore orientations. The sponges with unidirectional, parallel pores had higher tensile strength, Young’s modulus and swelling capacity than their multidirectional counterparts. Furthermore, ectopic bone formation was significantly increased in the unidirectional sponges when loaded with BMP-2 and intramuscularly implanted in rats, with a parallel arrangement of the new-formed bone trabeculae, resembling the disposition found in cortical bone (yet unpublished) [63].

## 4. Crosslinking of Collagen Type I-Based Biomaterials

Collagen type I is extremely abundant in nature, easy to isolate and highly soluble in acidic solutions. This has allowed for the fabrication of a great variety of different collagen scaffolds for tissue engineering, such as collagen sponges, tubes, sheets, hydrogels, injectable solutions, nanoparticles, pellets, or tablets. The manufacturing and details of their fabrication processes have been reviewed previously [64,65,66]. In the specific field of BTE, one of the characteristics an ideal biomaterial must possess is a degradation rate in accordance with the time needed for the patient’s body to form biomechanically stable new bone. 

Osteogenesis is a complex, multifactorial process that requires a significant amount of time. After a first inflammatory stage, characterized by the expression of pro-inflammatory cytokines and in which macrophages, lymphocytes, and other cells from the immune system invade the implant site, progenitor cells will migrate and differentiate within the implant to start the first stages of the osteogenic phases. The implanted biomaterial must remain undegraded, at least during these initial phases, to act as a support for the cells to proliferate and/or differentiate on. However, polymeric collagen type I is naturally very susceptible to biodegradation as it is a target for multiple collagenase enzymes that are being expressed by a variety of cells. Therefore, one very important step during the manufacturing of collagen-based scaffolds is crosslinking to reduce the natural biodegradation process and to increase the mechanical strength and stability by establishing intermolecular bonds [67]. Furthermore, crosslinking of collagen has shown to increase its capacity to support angiogenesis [68]. It is well known that osteogenesis is strictly dependent on successful vascularization of the implants and achieving this is one of the greatest challenges of modern BTE [69]. Although collagen type I itself already stimulates angiogenesis in vitro and in vivo [70], different strategies have been tested for increasing the vascularization of collagen-based biomaterials [71,72]. 

Crosslinking of collagen materials can be achieved using physical methods, such as dehydrothermal treatments [73,74] or ultraviolet light irradiation [75]. Since these methods are toxin-free, they could be considered very suitable for crosslinking collagen for biomedical use. However, physical crosslinking methods can only lead to a mild crosslinking between the collagen fibers, which is generally insufficient to prevent fast degradation in vivo. Hence, collagen scaffolds used for BTE are mostly crosslinked using chemical treatments, such as glutaraldehyde, isocyanates, or carbodiimides [76,77,78]. Although these methods are able to lower the degradation rate of collagen scaffolds enough to allow neo-osteogenesis within them, they are cytotoxic and non-reacted or side-produced residual molecules that remain in the scaffold might trigger undesired effects after implantation, such as inflammation or encapsulation [78,79]. The main methods for crosslinking collagen scaffolds have been recently analyzed by Adamiak & Sionkowska [67] and are summarized in Table 2.

To avoid some of the problems related to the use of high concentrations of chemical crosslinkers, we have recently fabricated a collagen type I biomaterial for BTE in the form of sponge-like scaffolds, which were crosslinked with a combination of dehydrothermal and chemical glutaraldehyde crosslinking methods [80]. This allowed us to use a concentration of glutaraldehyde significantly lower than the ones generally used. In addition, these sponges were produced from purified, bovine atelocollagen. Frequently, collagen-based biomaterials used for BTE are fabricated using polymeric collagen extracted from animal tissues and the general tendency is to keep these molecules as close to their native conformation as possible. However, these collagen fibrils keep N- and C-terminal telopeptides, which have been shown to be responsible for most of the immune reactions reported against collagen [81]. Hence, the use of purified atelocollagen, together with the combined physical and chemical crosslinking, might help overcome some of the drawbacks of many collagen sponges used currently in BTE approaches. 

**Figure 3:**
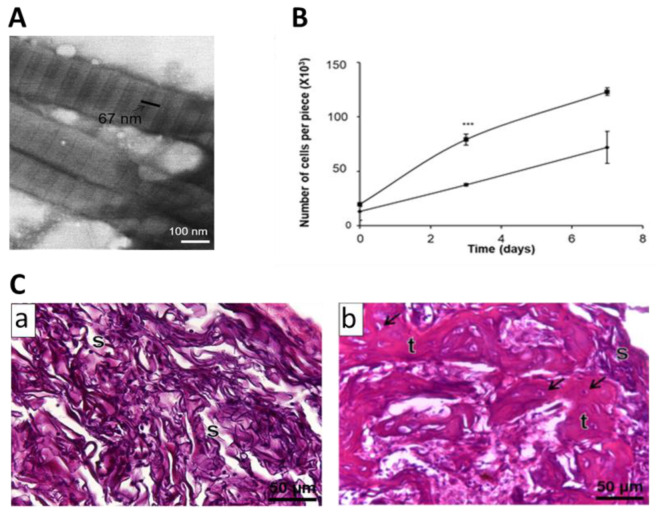
Characterization of a double-crosslinked atelocollagen sponge. (**A**) STEM images of negatively stained atelocollagen nanofibrils, showing an axial periodicity of 67 nm. (**B**) Adhesion and proliferation of MC3T3-E1 preosteoblasts on double-crosslinked atelocollagen sponges (DColS-0.0015G, ⯀) or atelocollagen sponges only crosslinked by a dehydrothermal method (DColS, ♦). Mean ± standard deviation. *** *p* < 0.001. (**C**) hematoxylin-eosin staining of histological sections of intramuscular ectopic implants. DColS (**a**) and DColS-0.0015G (**b**) sponges were loaded with 600 ng of BMP-2 and implanted for 21 days. Only the DColS-0.0015G scaffolds were able to support osteogenesis. **s**: collagen; **t**: bone trabeculae; **arrows**: osteocytes. Modified with permission from [80].

An extensive physicochemical characterization of the sponges, as well as osteoprogenitor cell adhesion, proliferation, and differentiation tests in vitro were performed. Finally, intramuscular implantation of these sponges loaded with BMP-2 demonstrated that they supported ectopic osteogenesis in vivo (Figure 3). These data indicated that these sponges might be a suitable alternative to sponges made from collagen directly extracted from animal tissues.

## 5. Biological Functionalization of Collagen Type I Biomaterials

Although collagen type I fibers contain multiple biologicals cues that can directly interact with cells to modulate their adhesion, proliferation and/or differentiation [82], collagen alone is not osteoinductive per se. Its excellent osteoconductive properties might have been confused with osteoinduction from time to time, but there is no proof that collagen alone can induce bone formation in a non-osseous environment without the influence of external signals. Nevertheless, collagen-based biomaterials can be modified to include other bioactive domains to direct the cells that interact with them towards certain differentiation pathways. The so-modified collagen biomaterial could be considered as osteoinductive if the proper biological cues are added.

Several strategies can be used for adding bioactive molecules to a protein-based biomaterial, and the strategy of choice should depend on different factors, such as: (i) if the added molecule needs to remain attached to the biomaterial or should be released at a certain rate instead; (ii) the mechanism of action of the added bioactive molecule; and (iii) the degradation rate of the biomaterial. 

Besides functionalization of biomaterials with specific chemicals or drugs, which will not be detailed here but has been extensively reviewed by Maia and co-workers [83], the most common molecules used for adding bioactive features to a biomaterial are growth factors, natural peptides, or synthetic peptides which sequence derives from the receptor-interacting domain of a growth factor or cytokine. 

### 5.1. Adsorption

**Figure 4:**
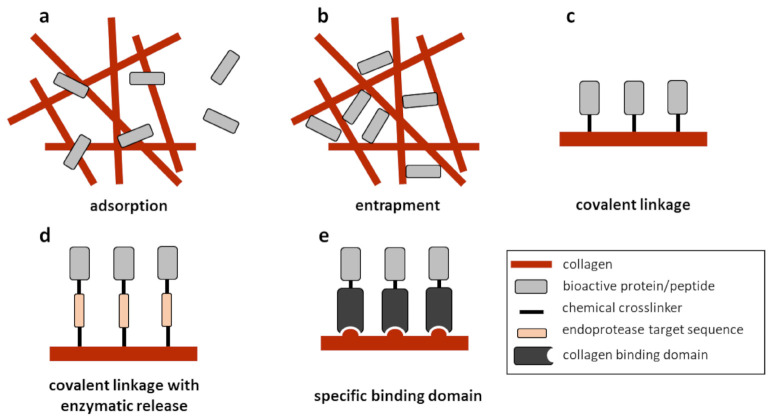
Schematic representation of different approaches for administering bioactive molecules in/on a collagen-based biomaterial for tissue engineering purposes: adsorption (**a**), entrapment (**b**), covalent linkage (with or without controlled release, (**c**,**d**) or specific binding through collagen binding domains (**e**).

The simplest strategies imply adsorbing bioactive proteins or peptides directly to the processed biomaterial (Figure 4a). In this case, the binding will mostly depend on the natural electrochemical complementarity between the added molecule and collagen. However, simple adsorption will usually lead to a quick bulk release which will only be suitable for molecules that are naturally soluble in vivo and must interact as such with the cell receptors.

Adsorption is currently the main strategy for delivering bioactive molecules in clinical contexts of BTE. Collagen type I biomaterials have been loaded with BMPs for BTE ever since their discovery. As early as 1991, a telopeptide-depleted bovine skin collagen was used as a carrier for a partially purified BMP fraction obtained from an osteosarcoma [84]. Parallel efforts were made to refine the already studied BMP fractions and, in the early 1990s the genes of BMP-2 [85] and BMP-7 [86] were cloned and expressed. These authors showed the great ability of these growth factors to induce bone formation when administered adsorbed to polymeric collagen sponges. As a result of all this research and the associated clinical trials [87], the American Food and Drug Administration (FDA) and the European Medicines Agency (EMA) approved the clinical use of BMP-2 loaded on bovine type I collagen biomaterials for intervertebral fusions, tibial open fractures, and maxillofacial procedures (INFUSE^®^, Medtronic). By the same date, also BMP-7 loaded onto a particulate collagen scaffold (Osigraft^®^/OP-1 Implant, Stryker) was approved for the treatment of non-unions in long bones [88]. However, given that osteogenesis is a very complex, multifactorial process in which different cell types and a great variety of molecular signals are involved, growth factors and cytokines other than BMPs can be added to collagen scaffolds to improve the osteoinductivity of these systems. Molecules such as FGF-2 have been used to enhance the osteoinductive activity of BMP-2 [89] and BMP-6 [90] and these effects were attributed to the induction of cell proliferation and to the pro-angiogenic activity of FGF-2. Collagen-based biomaterials have also been loaded with BMPs in combination with epidermal growth factor (EGF) [91], platelet-derived growth factor-BB (PDGF-BB) [92], insulin-like growth factor-1 (IGF-1) [93], or vascular endothelial growth factor (VEGF) [94,95] and in all these examples a clear and significant improvement in the respective bone regeneration models was reported in comparison to the administration of the BMP alone.

### 5.2. Entrapment

A second strategy consists of entrapping the growth factor or peptide in the biomaterial during its processing (Figure 4b). This is particularly interesting for molecules that need to be released at a sustained rate as the biomaterial is being degraded and is a frequently used strategy [96]. However, this approach might not always be suitable for collagen-based materials, as collagen type I is a supramolecular polymer obtained by multiple assembly steps through which the α-chains of collagen form fibrils and these arrange into fibers [11]. The addition of an exogenous molecule that will interact with the collagen molecules might interfere with fibrillogenesis or with collagen fiber formation during processing, resulting in a less organized structure. On the other hand, the fabrication of collagen sponges or hydrogels in vitro usually starts with an acid suspension of collagen molecules [80] and these conditions are not always suitable for adding complex growth factors that might degrade or precipitate at low pH or when it is neutralized to induce the fibrillogenesis. 

Furthermore, the later crosslinking procedures to stabilize the collagen scaffold might irreversibly inactivate the biological activity of the peptide or growth factor, as the crosslinking agents will form covalent bonds between the latter and the collagen fibers [96]. 

### 5.3. Covalent Linkage

Another option consists of covalently linking the added molecule to the collagen fibers (Figure 4c), which can be achieved by using a great variety of chemical reactions [83]. In this case, a more robust binding to the biomaterial is obtained and no spontaneous release of the protein or peptide will occur. This is especially interesting when integrin-binding molecules are used for functionalization, as integrins are involved in establishing interactions between the cells and their environment in a process in which mechanosensing plays a crucial role. When the added molecule is a naturally soluble growth factor or cytokine, it should be taken into account that the chemical processing needed for covalent linking might irreversibly affect the bioactive factor, causing a loss of its activity [97]. 

If release of the molecule must occur, but this should happen at a specific time point within the bone formation process, a controlled release strategy must be designed [98]. By inserting a biomolecule-sensitive cleavable element between the protein/peptide and the collagen fibers, a controlled release of the bioactive molecule can occur when a specific biomolecule is expressed in situ (Figure 4d) [83,99]. Although not exactly the same, a somewhat similar approach was tested when a matrix metalloproteinase (MMP)-cleavable crosslinker was used to attach BMP-2 to a PEG-based hydrogel. When implanted into a cranial bone defect in rats, cell migration eventually led to a local MMP secretion, causing the hydrogel to degrade and to release the BMP-2 molecules entrapped inside [100]. 

### 5.4. Specific Binding through Affinity Domains

A slightly more sophisticated strategy consists of designing and producing chimeric proteins or peptides that include specific binding domains to non-covalently attach to a biomaterial with high affinity (Figure 4e). In these cases, no chemical modifications of the scaffold, peptide/growth factor or both are needed, and the release rate of these molecules will depend on both the specific affinity of the binding domain to the biomaterial and on the degradation rate of the latter. In the specific case of collagen, several different domains have been described and used to produce chimeric molecules, such as the collagen binding domain (CBD) found in the von Willebrand factor (vWF; WREPSFCALS) or that from the collagenase of *Clostridium histolyticum* (TKKTLRT) [101]. Growth factors such as TGF-β1 [102,103], FGF-2 [104], PDGF [105], or VEGF [106] have been successfully modified to incorporate collagen-binding domains.

**Figure 5:**
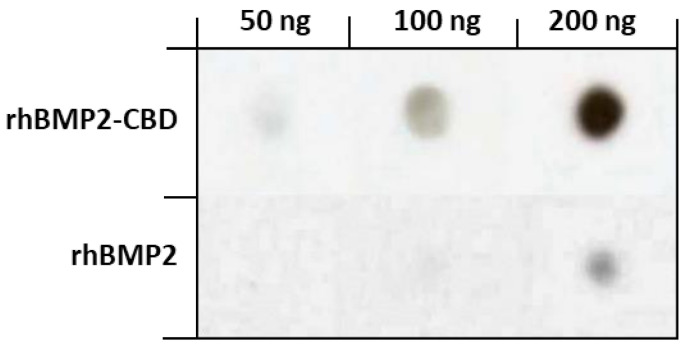
Collagen binding of a rhBMP2-CBD chimeric growth factor. Collagen type I sponges were loaded with different amounts of rhBMP-2 or rhBMP2-CBD, let dry and washed with PBS for 7 days. Molecules that remained bound to the sponges were detected using an anti-BMP-2 antibody. Modified from [107].

In this line, we designed and produced a chimeric rhBMP-2 with a CBD modified from the vWF at the N-terminus of the BMP monomers using an *E. coli*-based expression system [107]. This so-called rhBMP2-CBD was able to bind to collagen type I sponges with higher affinity than the unmodified rhBMP-2, and to remain in the sponges after at least 7 days of extensive washing with phosphate buffered saline (Figure 5). Furthermore, the biological activity of rhBMP-2CBD measured in vitro was similar to that of rhBMP-2; however, when loaded on a collagen type I sponge and implanted into muscular pouches in rats, rhBMP2-CBD was able to induce ectopic bone formation at a lower dose than rhBMP-2. This indicated that the collagen sponge functionalized with the chimeric growth factor had become an osteogenic biomaterial capable of steadily releasing BMP-2 molecules.

It has been shown that BMP-6 has a stronger osteoinductive activity than BMP-2 [93,108] and with this in mind, a similar approach was used to attempt the production of a chimeric rhBMP6-CBD. Unfortunately, it was shortly after demonstrated that, contrarily to what happens with BMP-2, the binding of BMP-6 to its receptor is strictly dependent on the N-glycosylation of the growth factor [109], making it impossible to produce active BMP-6 molecules in basic *E. coli* expression systems (unpublished data). Further attempts to produce the rhBMP6-CBD chimera in baculovirus-transfected insect cells were also unsuccessful (unpublished data). This illustrates the fact that this strategy is not suitable for any cytokine or growth factor, as the addition of new sequences to the primary structure of a protein might interfere with its expression, folding, or biological activity.

**Figure 6:**
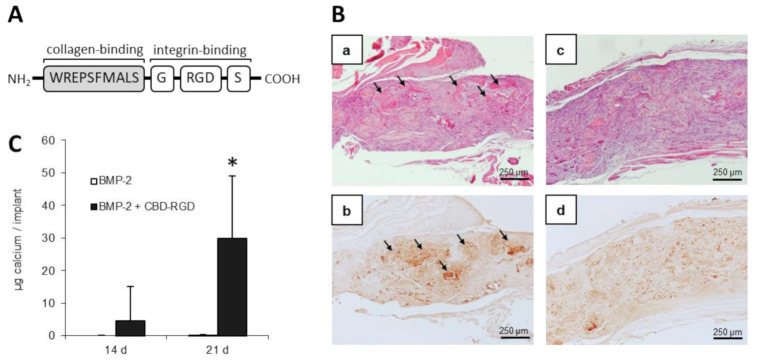
Biological activity of a collagen binding domain-Arg-Gly-Asp (CBD-RGD) synthetic peptide. (**A**) Schematic representation of the CBD-RGD peptide. (**B**) Ectopic bone formation in rats. Collagen type I sponges were functionalized with the CBD-RGD peptide (**a**,**b**) or not (**c**,**d**), loaded with 300 ng of BMP-2 and intramuscularly implanted in rats for 21 days. Upper images (**a**,**c**) are hematoxylin-eosin stained sections of the implants showing mature bone trabeculae in the sponges that had been functionalized with CBD-RGD. Lower images (**b**,**d**) correspond to anti-osteopontin immunohistochemistry. Arrows: bone trabeculae containing osteoblasts. (**C**) Calcium content of the recovered ectopic implants. No detectable calcium could be found within the non-fuctionalized implants. Contrarily, a significant amount of calcium was measured in the ectopic implants after 21 days. * *p* ≤ 0.01. Modified from [113].

As said earlier, besides entire growth factors, naturally occurring or synthetic bioactive peptides can also be used for functionalizing collagen. There are dozens of peptides described with interest in BTE, either mediating cell adhesion, proliferation, or differentiation [110]. The most studied peptides are those containing the integrin-binding Arg-Gly-Asp (RGD) motif. This tripeptide, which was identified over three decades ago as the minimal cell adhesion sequence found in fibronectin [111], can bind to multiple integrins to promote cell adhesion and/or differentiation. Besides RGD, collagen type I contains several motifs that have been shown to mediate its interaction with cell’s integrins, such as GFOGER [112] or DGEA [16]. However, the RGD tripeptide is apparently not exposed in native collagen, but only becomes accessible in partially denatured fibers [15]. With this in mind, we designed a synthetic peptide that contained a modified vWF-derived CBD and the consensus RGD sequence, with the aim of functionalizing collagen type I scaffolds to increase the adhesion and/or osteoblastic differentiation of osteoprogenitor cells [113]. When loaded onto absorbable collagen type I sponges, approximately 1 µg of CBD-RGD peptide was able to remain bound to 1 mg of collagen and the binding was stable for at least 7 days. This so-called CBD-RGD peptide (Figure 6) increased the expression of the osteoblastic marker enzyme alkaline phosphatase (ALP) by murine preosteoblastic MC3T3-E1 cells cultured on the sponges. Furthermore, when collagen scaffolds functionalized with the CBD-RGD peptide were loaded with rhBMP-2 and implanted into muscular pouches in rats, ectopic osteogenesis was observed with as little as 300 ng of BMP-2, which is known to be a subfunctional dose of this growth factor.

## 6. Composite Materials with Collagen Type I

Although collagen has some excellent properties that justifies its use in BTE applications, no biomaterial can be considered as ideal. The complexity of the molecular interactions and signals found in bone tissue cannot be mimicked by the use of collagen alone, since the cells that play a role in osteogenesis and bone homeostasis receive information from a large amount of bone constituents, including soluble factors and the organic and inorganic molecules that form the ECM of bone. Furthermore, although the physical and biomechanical properties of collagen biomaterials can be modified to a certain extent [67], they cannot meet the optimal requirements for BTE purposes. Hence, collagen is frequently used in combination with other biomaterials to form composites [17,114].

Since apatites are the main inorganic component of the bone ECM, different forms of hydroxyapatite have been combined with collagen type I to obtain composite biomaterials with the advantages of both constituents. In these cases, HA provides its excellent biomechanical and biological properties, while collagen helps reducing the fragility of the HA scaffold and enriches the biological activity of the composite with its bioactive domains. The mechanical strength of collagen-based scaffolds can be increased by incorporating HA and controlling key parameters, such as the concentration of collagen, the amount and type of HA added, and the crosslinking method used [115]. Furthermore, the roughness that HA nano or microparticles provide to a collagen scaffold will positively affect cell proliferation and differentiation [116]. More detailed information about collagen-apatite composite biomaterials can be found in the review by Kołodziejska et al. [117]. Other bioceramic components that have been extensively explored to form composites with collagen are β-tricalcium phosphate (β-TCP) and bioglasses, which have all shown to improve the mechanical properties of collagen-based scaffolds and to increase osteoblastic cell differentiation [17].

Collagen-containing composites can also be obtained with other natural polymeric materials, such as glycosaminoglycans (GAGs), silk fibroin (SF), or chitosan. SF, obtained from the silk produced by certain arthropods, has remarkable mechanical properties and a tunable degradation rate, what makes it a very interesting scaffolding material for BTE [118]. Nevertheless, the biomaterial-cell interaction can be significantly improved when SF is combined with collagen, which enhances osteoblastic cell attachment, growth, and differentiation [119]. More complex approaches involve combining more than two biopolymers, such as collagen, fibroin, and chitosan. A comparative analysis with nine different combinations of these polymers revealed their different mechanical properties and that the best growth of MG-63 cells was obtained when collagen and fibroin were mixed at a 50:50 proportion and 20% chitosan was added to the final product [120].

Besides natural polymers, collagen can also be combined with synthetic polymeric materials, such as PLA, PGA, PLGA, polycaprolactone (PCL), or poly (vinyl alcohol). All these polymers are biocompatible and biodegradable, and have been used widely in BTE applications. However, most of them are rather hydrophobic, what limits the interactions between cells and the scaffold and, hence, their use in pure form. Therefore, synthetic polymers are frequently used in combination with more bioactive materials, such as collagen. In these cases, the synthetic polymer is commonly used to generate a macroporous backbone in which collagen creates a microporous environment for the cells to nest in and to promote their differentiation [121].

More in-depth information on collagen-containing composite scaffolds for bone regeneration can be found in the review written by Zhang et al. [17].

## 7. Conclusions

Collagen type I, either directly extracted from animal sources, purified, or produced as recombinant proteins, has been used for decades in orthopedic surgery and in bone tissue engineering research. Although collagen-based biomaterials fail in providing the mechanical support that is generally needed in bone regeneration scenarios, the excellent biological properties of collagen type I and its great versatility make it a highly valuable component in BTE strategies. Future research in this field should point to reduce the costs of recombinant or purified atelocollagen to avoid the immunogenicity, batch-to-batch variability and risks associated to the zoonotic transmission of diseases of collagen obtained directly from animal sources. Furthermore, exploring new ways to improve the mechanical properties and to tailor the degradation rate of collagen-based biomaterials would be useful to expand their applications. Also, the combination of collagen with new biomaterials, as well as the rational modification or functionalization of collagen with bioactive peptides or chemicals could lead to BTE scaffolds that better mimic the complexity of native bone tissue. Modern techniques and nanotechnology will allow for the use of collagen type I in new designs that might better comply with all the requirements that a biomaterial should have to be considered ideal.

## Figures and Tables

**Table 1:** Different forms of collagen-based biomaterials for several tissue engineering applications. CBD-FGF-2, collagen binding domain-FGF-2; ucMSC, umbilical cord-derived mesenchymal stem cells; HA, hydroxyapatite; PLGA, poly(lactic-co-glycolic)acid; PGA, poly(glycolic acid); BMP, bone morphogenetic protein.

Collagen Biomaterial Form	Combination/Modification	Biological Model	Main Results	References
Powders/particles	CBD-hFGF-2	Mouse femur fracture	Increase of callus volume and bone mineral content	[33]
	CBD-rhBMP-2	Vertebral laminar defects in rabbits	Greater bone regeneration. Signs of bone formation even without growth factor	[34]
	ucMSC	Rabbit alveolar cleft model	Formation of a significant amount of new bone, higher percentage of bone trabeculae but no more mineral density	[35]
Fibers and tubes	-	Rat tibial nerve resection	Evidences of some degree of histological regeneration at surgical site	[36]
	UV irradiation crosslinking	Rat sciatic nerve section	Space of the tube is preserved and nerve repair was comparable to isograft treatment	[37]
	BMSC	Mouse sciatic nerve section	Scaffolds loaded with cells induced better regeneration of peripheral nerve fibers	[38]
	Electrospun in combination with HA	*In vitro* cell viability and osteogenic differentiation assay	Good physicochemical properties and feasible manufacturing process. U2-OS cells remain viable and differentiate to osteoblast	[39]
	Electrospun with PLGA and HA nanorods	*In vitro* cell viability and osteogenic differentiation assay	MC3T3-E1 cells proliferate on the scaffold. Osteogenic differentiation is evidenced by different markers	[40]
	Electrospun collagen containing catecholamines and Ca^2+^	*In vitro* human foetal osteoblasts viability and osteogenic differentiation	Good mechanical properties. Bio-inspired in situ chemical crosslinking and mineralization strategy. Osteogenic differentiation is evidenced by different markers	[41]
	Electrospun in combination with chitosan	Rat full-thickness cranial defects	Composite had improved physicochemical properties and induced almost a total regeneration 8 weeks after implantation	[42]
Gels	HA particles and bone marrow cells	Rabbit posterolateral lumbar spine fusion model	Homogeneous new trabecular bone formation similar to autograft and BMP-HA group.	[43]
	Adipose-Derived stem cells + PLGA-β-TCP scaffold	Rabbit intramuscular ectopic bone formation assay	Composites showed new bone formation evidenced by radiography, histology and histomorphometric bone occupation analysis	[44]
	rhBMP-2	Rabbit tendon-bone interface injury model	Collagen-BMP-2 gel increased fusion rate between the bone tunnel and tendon	[45]
	3D printed in combination with HA	*In vitro* viability and proliferation of Vero cells	Superior control over scaffold morphology and porosity. Supernatants of these gels incubated in medium were not cytotoxic	[46]
	3D printed collagen containing rod-like nano-HA	-	Highly controlled 3D printed mesh-like structures with a homogeneous HA distribution.	[47]
	3D printed in combination with decellularized extracellular matrix and silk fibroin	*In vitro* MC3T3 viability and osteogenic differentiation	Feasible hybrid 3D printing method. Cell proliferation and osteogenic differentiation was higher in comparison with only-collagen controls.	[48]
Sponges	PGA + AD-MSC	Rabbit calvarial bone defect	Significant improvement of bone formation by CT scan imaging analysis induced by scaffolds with or without cells.	[49]
	Bone marrow mononuclear cells + Nano- Hydroxyapatite + platelet-rich fibrin	Human patients with unilateral alveolar cleft defects	Patients exhibited less donor site complications, faster and better soft tissue healing, less postoperative pain and a higher rate of complete alveolar bone union.	[50]
	Precultured system of mesenchymal stem cells + mechano-chemical induction	Rat Achilles tendon repair model	Improved tenogenic differentiation *in vitro*. In vivo improvements in Achilles functional index, Young’s modulus and histology score	[51]
	Umbilical cord mesenchymal stem cell-derived nanovesicles + rhBMP-2	Nude mouse calvarial defect model	Micro-CT imaging analysis evidenced increased bone volume and number of trabeculae. Histology revealed increased number of vessel structures.	[52]

**Table 2:** Main methods for crosslinking collagen, according to [67].

Type of Crosslinking		Main Characteristics
**Chemical**	Glutaraldehyde (GA)	Low cost. High reactivity. High water solubility. Cytotoxic.
	Genipin	Less toxic than other chemical crosslinkers. Might promote osteoblastic differentiation. Not suitable for gelatin crosslinking.
	1-Ethyl-3-(3-dimethylaminopropyl) carbodiimide (EDC) and N-hydroxysuccinimide (NHS).	Zero-length crosslinker. Acts mainly as an intra-fibrillar crosslinker. Low cytotoxicity.
	Dialdehyde starch	Can be used for intra- and intermolecular crosslinking. Low cytotoxicity. Biodegradable. Has antiviral activity.
	Chitosan	Biodegradable. Non-toxic. Has antibacterial and antifungal activity. Poor water solubility. Tends to form polydisperse solutions.
**Physical**	Dehydrothermal (DHT) treatment	Non-toxic. Provides sterilization. May cause collagen denaturation.
	UV light	Faster than DHT. Non-toxic. Provides sterilization. May cause collagen denaturation.
**Enzymatic**	Microbial transglutaminase	Similar to natural crosslinking. More expensive.

## Data Availability

The data presented in this study are available on request from the corresponding author.
